# Zone-Specific PSA Density from AI-Assisted mp-MRI as a Predictor of Prostate Cancer in the PSA 4–20 ng/mL Range

**DOI:** 10.3390/jcm15155769

**Published:** 2026-07-23

**Authors:** Abdullah Golbasi, Huseyin Bicer, Ali Yasin Ozercan, Burak Elmaagac, Omer Sahin, Mert Ali Karadag

**Affiliations:** 1 Department of Urology, Kayseri City Hospital, University of Health Sciences Medical Faculty of Kayseri, Kayseri 38280, Türkiye; 2 Department of Urology, Kayseri City Hospital, Kayseri 38280, Türkiye

**Keywords:** Prostate cancer, PSA density, Transition zone, Multiparametric MRI

## Abstract

**Background/Objectives:** Serum prostate-specific antigen (PSA) lacks sufficient specificity for prostate cancer (PCa) detection. We evaluated the diagnostic value of AI-assisted MRI-derived zone-specific PSA density parameters, including transition zone PSA density (TZ-PSAD) and peripheral zone PSA density (PZ-PSAD), in men undergoing prostate biopsy. **Methods:** This retrospective cohort study included 354 patients who underwent prostate biopsy due to elevated PSA (2024–2026), stratified into PSA 4–10 ng/mL (*n* = 272) and PSA 10–20 ng/mL (*n* = 82) groups. Zonal volumes were measured using AI-assisted mp-MRI segmentation per PI-RADS v2.1, and PSAD, TZ-PSAD, and PZ-PSAD were calculated accordingly. MRI readings were performed blinded to pathology. **Results:** TZ-PSAD was significantly higher in PCa patients in both PSA groups (*p* < 0.001), demonstrating superior AUC compared to PSAD in both the PSA 4–10 ng/mL (0.711 vs. 0.660) and PSA 10–20 ng/mL (0.841 vs. 0.676) groups. At an optimal cut-off of ≥0.34 ng/mL^2^, TZ-PSAD yielded 44.8% sensitivity and 90.8% specificity in the PSA 4–10 group, improving to 73.8% sensitivity and 94.7% specificity at ≥0.48 ng/mL^2^ in the PSA 10–20 group. On multivariate analysis, TZ-PSAD and PI-RADS ≥ 3 emerged as independent predictors of PCa in both groups. Serum PSA, PZ-PSAD, and PZ volume failed to differentiate BPH from PCa in either group. **Conclusions:** TZ-PSAD and conventional PSAD were both significantly associated with prostate cancer, whereas PZ-PSAD was not, and TZ-PSAD, combined with PI-RAD ≥3, emerged as an independent predictor in the PSA gray zone.

## 1. Introduction

Prostate cancer (PCa) ranks among the most prevalent malignant neoplasms in men, with both its incidence and diagnosis rate showing a steady increase in recent years. Prostate-specific antigen (PSA) has long been the most widely used serum biomarker in the diagnostic workup of PCa. However, as PSA is an organ-specific rather than a cancer-specific marker, elevated levels may also be observed in non-malignant conditions such as prostatitis and benign prostatic hyperplasia (BPH) [1].

This situation significantly limits the diagnostic specificity of PSA and leads to unnecessary prostate biopsies. For this reason, additional diagnostic parameters have been developed to complement PSA [2]. Studies have shown that prostate-specific antigen density (PSAD), calculated by dividing the PSA value by total prostate volume, may offer higher diagnostic accuracy in detecting prostate cancer compared to PSA alone [3].

The ability to reliably measure peripheral zone (PZ) and transitional zone (TZ) volumes separately using artificial intelligence-assisted multiparametric magnetic resonance imaging (mp-MRI) has made zone-specific PSA density calculations clinically feasible. This advancement has strengthened the hypothesis that zone-specific PSA density parameters, namely peripheral zone PSA density (PZ-PSAD) and transitional zone PSA density (TZ-PSAD), may offer superior diagnostic accuracy over conventional PSAD in distinguishing prostate cancer from BPH. In this study, we aimed to investigate the clinical value of TZ-PSAD and PZ-PSAD in the diagnosis of prostate cancer among patients who underwent prostate biopsy with serum PSA levels in the 4–10 ng/mL and 10–20 ng/mL ranges.

## 2. Materials and Methods

### 2.1. Study Design and Patient Population

**Figure 1:**
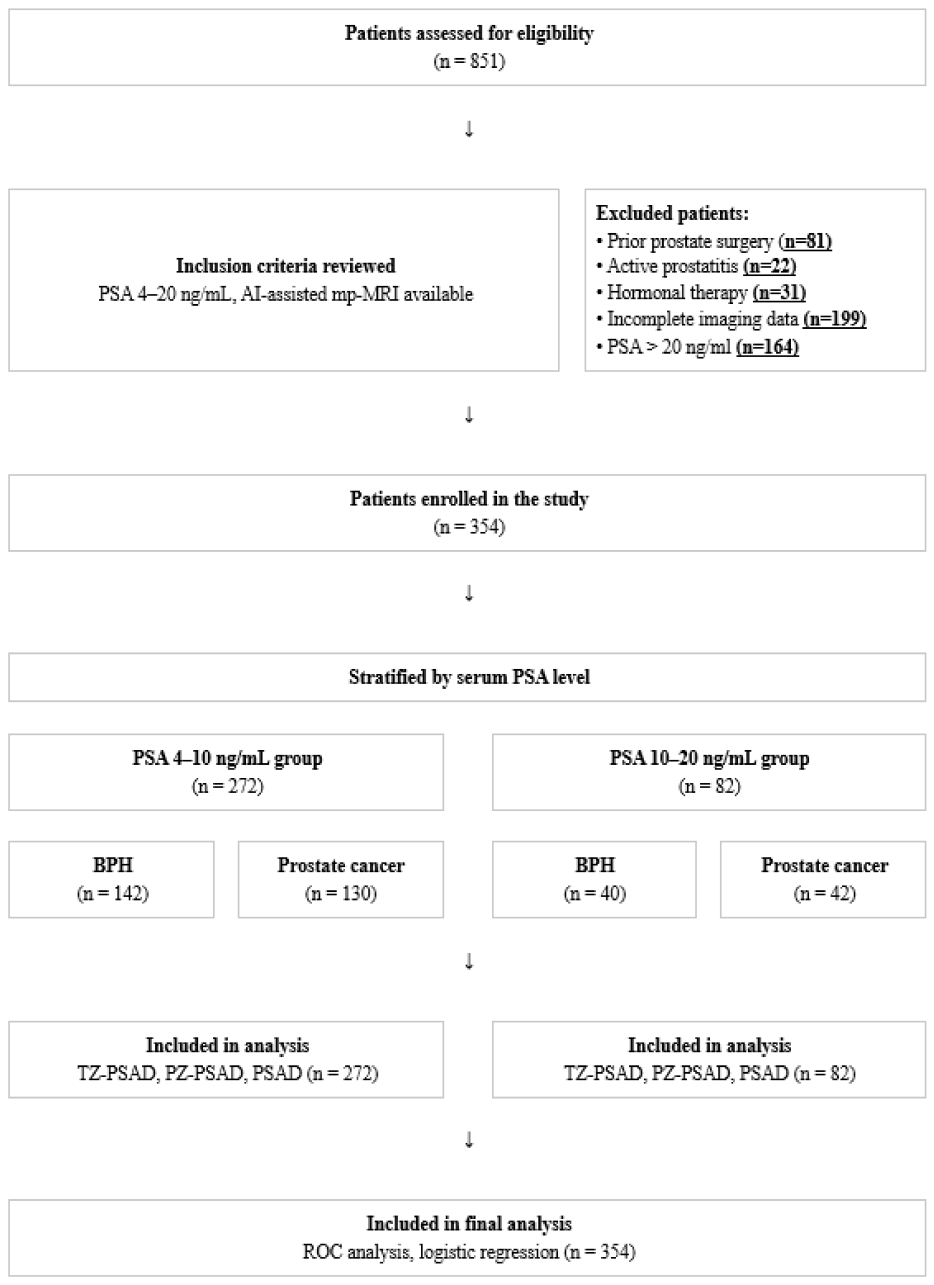
Patient flow diagram. **Abbreviations:** mp-MRI: multiparametric magnetic resonance imaging; AI: artificial intelligence; TZ-PSAD: transition zone PSA density; PZ-PSAD: peripheral zone PSA density; PSA: prostate-specific antigen; BPH: benign prostatic hyperplasia; ROC: receiver operating characteristic.

This retrospective cohort study was conducted at our institution between 2024 and 2026. The study population comprised consecutive patients who underwent transrectal prostate biopsy following mp-MRI evaluation due to elevated serum PSA levels. All patients underwent combined biopsy, consisting of cognitive MRI-targeted biopsy of any PI-RADS ≥ 3 lesion (3–4 cores per index lesion, obtained by cognitive fusion between the mp-MRI findings and real-time transrectal ultrasound) followed by a 12-core systematic biopsy in the same session. Patients with more than one PI-RADS ≥ 3 lesion underwent targeted sampling of each lesion in addition to the systematic cores. The biopsy protocol was applied uniformly across the study period and did not differ according to PI-RADS category, aside from the additional targeted cores obtained in patients with a PI-RADS ≥ 3 lesion. Patients were stratified into two groups based on serum PSA concentration: the PSA 4–10 ng/mL group (BPH: *n* = 142, PCa: *n* = 130) and the PSA 10–20 ng/mL group (BPH: *n* = 40, PCa: *n* = 42). Inclusion criteria were a serum PSA level between 4 and 20 ng/mL, availability of pre-biopsy mp-MRI with zone-based volumetric measurements, and histopathological confirmation of BPH or PCa on biopsy. Patients with prior prostate surgery, active prostatitis, hormonal therapy, PSA > 20 ng/mL, or incomplete imaging data were excluded. The patient selection process is illustrated in Figure 1. The study was conducted in accordance with the Declaration of Helsinki and institutional ethics committee approval was obtained.

### 2.2. AI-Assisted Evaluation of MRI Images

Prostate zonal segmentation (transition zone and peripheral zone) was performed using an MDR-CE-marked artificial intelligence system (hProstate v1.0, HeviAI, Istanbul, Turkey). According to the manufacturer, the underlying deep learning algorithm was developed and validated using a training dataset of approximately 7500 bi-parametric prostate MRI examinations, achieving an area under the curve (AUC) of 0.88 for the detection of clinically significant prostate cancer. The segmentation architecture is based on a self-adapting 3D nnU-Net framework that generates anatomically guided, fully automated zonal contours without requiring manual delineation or operator-dependent input, as described in the original development and multi-center validation study [4]. In the present study, all segmentations were generated automatically by the software and no manual correction of the AI-derived contours was performed. TZ and PZ volumes obtained from these segmentations were used to calculate TZ-PSAD and PZ-PSAD for each patient. Zone-specific PSA density parameters were subsequently calculated as follows: PSAD was derived by dividing serum PSA by total prostate volume; TZ-PSAD by dividing serum PSA by TZ volume; and PZ-PSAD by dividing serum PSA by PZ volume. All measurements are expressed in ng/mL^2^. PI-RADS scores were assigned by experienced uroradiologists blinded to biopsy outcomes.

### 2.3. Data Collection

Clinicopathological data were retrieved from prospectively maintained institutional records. The following variables were collected for each patient: age, comorbidities (hypertension, diabetes mellitus, coronary artery disease, and COPD), digital rectal examination (DRE) findings categorized as normal (Grade 1), mildly enlarged (Grade 2), or significantly enlarged (Grade 3), serum PSA level, mp-MRI PI-RADS score, total prostate volume, PZ and TZ volumes, PSAD, TZ-PSAD, PZ-PSAD, and biopsy histopathology. In patients with confirmed prostate cancer, the International Society of Urological Pathology (ISUP) grade group was additionally recorded.

### 2.4. Statistical Analysis

Continuous variables were tested for normality using the Kolmogorov–Smirnov and Shapiro–Wilk tests. As the majority of continuous variables exhibited non-normal distribution, data are expressed as median (interquartile range, IQR) unless otherwise specified, and between-group comparisons were performed using the Mann–Whitney U test. Categorical variables are presented as frequency (percentage) and were compared using the Pearson chi-square test.

Patients were stratified into two groups according to serum PSA level: PSA 4–10 ng/mL and PSA 10–20 ng/mL. Within each PSA stratum, patients were further classified as having benign prostatic hyperplasia (BPH) or prostate cancer (PCa) on histopathological examination, and comparisons were performed separately within each stratum.

Receiver operating characteristic (ROC) curve analysis was performed to evaluate the discriminative performance of prostate volume, transition zone volume, PSA density, and transition zone density for the detection of prostate cancer within each PSA stratum. The AUC with 95% confidence interval (CI) was calculated using the Hanley–McNeil method, and the optimal cut-off value for each parameter was determined using the Youden index (sensitivity + specificity − 1). As prostate volume and transition zone volume were inversely associated with the presence of prostate cancer, their discriminative values were assessed after directional inversion to enable AUC interpretation on a common scale (AUC > 0.5). Between-modality superiority of TZ-PSAD over conventional PSA density was additionally assessed using DeLong’s test for two correlated ROC curves, performed separately within each PSA stratum. To further assess whether the discriminative performance of TZ-PSAD reflects an incremental density effect beyond transition zone size alone, DeLong’s test was also used to compare TZ-PSAD versus transition zone volume alone in each PSA stratum.

Univariate logistic regression analysis was performed to identify factors associated with prostate cancer detection, including age, PSA, prostate volume, peripheral and transition zone volumes, PSA density, peripheral and transition zone densities, comorbidities (hypertension, diabetes mellitus, coronary artery disease, chronic obstructive pulmonary disease), DRE findings, and multiparametric MRI PI-RADS score. DRE findings were dichotomized as normal (Grade 1) versus enlarged (Grade 2–3) and PI-RADS score was dichotomized as PI-RADS 2 (reference) versus PI-RADS ≥ 3.

Variables achieving statistical significance (*p* < 0.05) on univariate analysis were entered into multivariate logistic regression using the forced-entry method. Because of substantial collinearity between volumetric and density parameters (Spearman ρ = 0.81–0.92), two separate multivariate models were constructed for each PSA stratum to avoid unstable parameter estimation: Model A (volumetric), incorporating prostate volume and transition zone volume (and age, where significant) together with significant categorical predictors; and Model B (density), incorporating transition zone density as the sole continuous density parameter together with significant categorical predictors. PSA density was excluded from Model B due to collinearity with transition zone density and its inferior discriminative performance on ROC analysis. Results of logistic regression are reported as (adjusted) odds ratios with 95% CI. To ensure clinically interpretable effect sizes and to address near-separation in the smaller PSA 10–20 ng/mL stratum, continuous density and volumetric predictors were rescaled to clinically meaningful increments (0.1 ng/mL^2^ for density parameters, 10 mL for volumes, 1 year for age), and all univariate and multivariable models were refitted using Firth penalized (bias-reduced) logistic regression. For the same collinearity-driven rationale used for Model B, Model A retained transition zone volume as the sole continuous volumetric predictor, since total prostate volume and transition zone volume were themselves highly collinear (Spearman ρ = 0.92).

As a complementary approach, forward stepwise logistic regression with the likelihood-ratio entry criterion (*p* < 0.05) was performed for each PSA stratum, incorporating all candidate continuous and dichotomized categorical variables. Model performance at each step was assessed using the overall correct classification rate and Nagelkerke R^2^; the step yielding the highest overall correct classification rate was selected as the final model.

A two-sided *p*-value < 0.05 was considered statistically significant for all analyses. Statistical analyses were performed using IBM SPSS Statistics, version 30 (IBM Corp., Armonk, NY, USA).

Internal validation of the final multivariable density model (Model B) in each PSA stratum was performed using bootstrap resampling (1000 replicates, Harrell’s optimism-correction method), yielding optimism-corrected estimates of the AUC and the calibration slope (shrinkage factor). As a pre-specified sensitivity analysis, ROC and DeLong comparisons were repeated using clinically significant prostate cancer (ISUP Grade Group ≥ 2) as the outcome, with ISUP Grade Group 1 reclassified together with BPH as the non-significant reference category. A decision-curve and biopsy-avoidance analysis was additionally performed at the Youden-optimal TZ-PSAD cut-off in each stratum, quantifying the proportion of any-grade and clinically significant cancers that would be missed if biopsy were deferred below this threshold.

## 3. Results

A total of 354 patients were included in the study. The PSA 4–10 ng/mL group included 142 patients with BPH and 130 with prostate cancer, while the PSA 10–20 ng/mL group included 40 patients with BPH and 42 with prostate cancer.

### 3.1. Patient Characteristics

No statistically significant difference in age was observed between BPH and prostate cancer patients in the PSA 4–10 ng/mL group (median 64.00 vs. 66.00 years; *p* = 0.059). Although comorbidity distribution was similar between groups, coronary artery disease was significantly more prevalent in BPH patients than in prostate cancer patients in the PSA 10–20 ng/mL group (27.5% vs. 7.1%; *p* = 0.031) (Table 1).

DRE findings differed significantly between BPH and prostate cancer in both PSA groups. In the PSA 4–10 ng/mL group, normal DRE findings (Grade 1) were more frequently observed in prostate cancer patients than in BPH patients (54.6% vs. 33.8%; *p* = 0.001), with a similarly significant difference noted in the PSA 10–20 ng/mL group (54.8% vs. 25.0%; *p* = 0.020) (Table 1).

PI-RADS score distribution differed significantly between prostate cancer and BPH in both groups (*p* < 0.001 for both). In the PSA 4–10 ng/mL group, a PI-RADS score of 5 was markedly more frequent in prostate cancer patients (18.5% vs. 3.5%). In the PSA 10–20 ng/mL group, this proportion reached 45.2% in prostate cancer patients compared to only 7.5% in BPH patients (Table 1).

### 3.2. Volumetric and PSA Density Parameters

Total prostate volume was significantly higher in BPH patients than in prostate cancer patients in both PSA groups (PSA 4–10 ng/mL: median 50.82 vs. 40.00 mL, *p* < 0.001; PSA 10–20 ng/mL: median 66.50 vs. 36.25 mL, *p* < 0.001). Transitional zone volume was similarly elevated in the BPH group (PSA 4–10 ng/mL: median 39.00 vs. 22.00 mL, *p* < 0.001; PSA 10–20 ng/mL: median 56.00 vs. 17.45 mL, *p* < 0.001). Peripheral zone volume, however, did not differ significantly between groups in either PSA group (*p* = 0.441 and *p* = 0.548). Serum PSA levels also failed to differentiate BPH from prostate cancer in either group (PSA 4–10 ng/mL: *p* = 0.526; PSA 10–20 ng/mL: *p* = 0.553). Standard PSAD was significantly higher in prostate cancer patients across both groups (PSA 4–10 ng/mL: median 0.18 vs. 0.13 ng/mL^2^, *p* < 0.001; PSA 10–20 ng/mL: median 0.30 vs. 0.21 ng/mL^2^, *p* = 0.006). TZ-PSAD emerged as the parameter providing the most pronounced discrimination in both PSA groups (PSA 4–10 ng/mL: median 0.29 vs. 0.18 ng/mL^2^, *p* < 0.001; PSA 10–20 ng/mL: median 0.68 vs. 0.22 ng/mL^2^, *p* < 0.001). PZ-PSAD, in contrast, did not achieve statistical significance in either group (*p* = 0.939 and *p* = 0.791) (Table 1).

### 3.3. ROC Analysis

**Figure 2:**
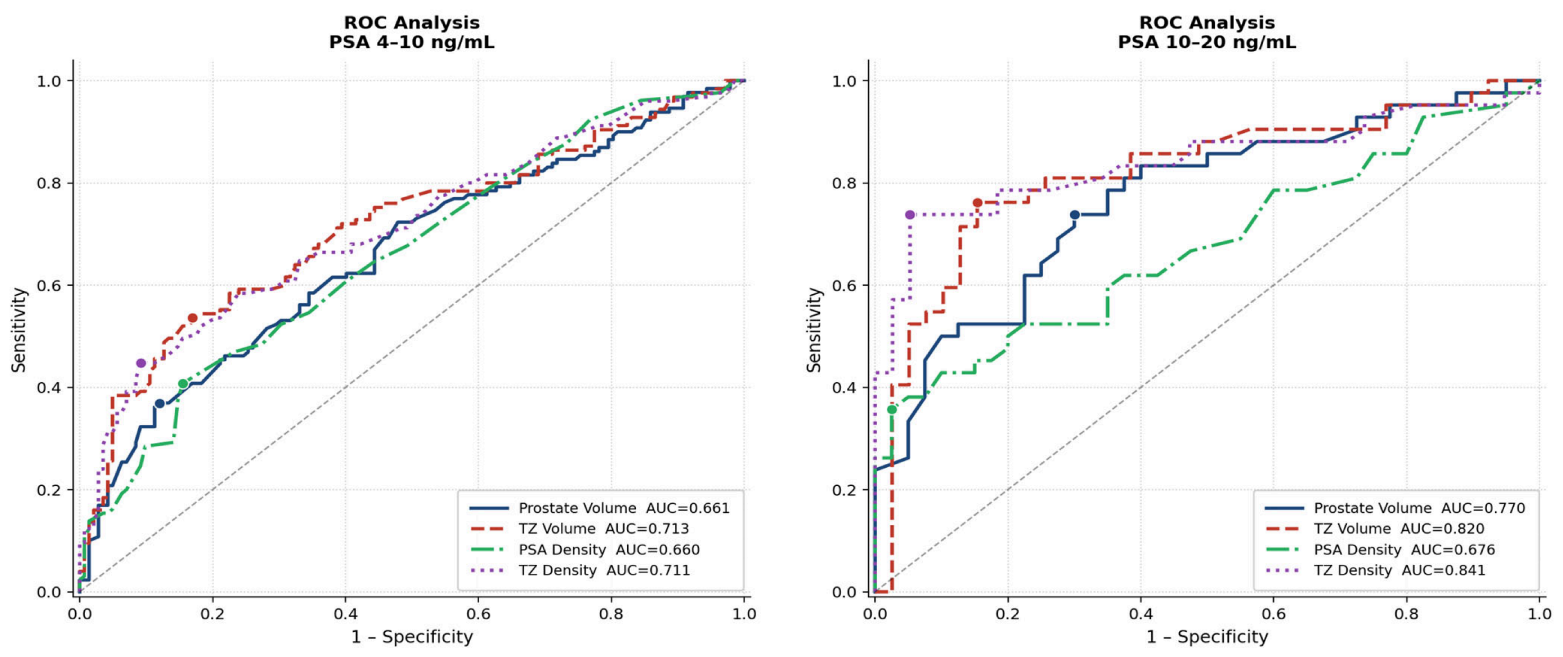
ROC curves for prostate cancer detection. Filled circles indicate the optimal cut-off point (Youden index) for each variable. PSA group 4–10 ng/mL (**left panel**); PSA group 10–20 ng/mL (**right panel**).

In the PSA 4–10 ng/mL group, TZ-PSAD (AUC = 0.711; 95% CI: 0.649–0.774) and TZ volume (AUC = 0.713; 95% CI: 0.651–0.776) provided the highest diagnostic accuracy for detecting prostate cancer. For TZ-PSAD, a cut-off value of ≥0.34 ng/mL^2^ yielded a sensitivity of 44.8% and a specificity of 90.8%. In the PSA 10–20 ng/mL group, TZ-PSAD demonstrated the highest diagnostic performance (AUC = 0.841; 95% CI: 0.754–0.928), providing strong discrimination with a sensitivity of 73.8% and a specificity of 94.7% at a cut-off value of ≥0.48 ng/mL^2^ (Figure 2). DeLong’s test confirmed that TZ-PSAD significantly outperformed conventional PSA density in both strata (*p* = 0.026 for the PSA 4–10 ng/mL group; *p* = 0.0002 for the PSA 10–20 ng/mL group; Table 2).

### 3.4. Logistic Regression Analysis

In the PSA 4–10 ng/mL group, univariate analysis identified TZ-PSAD (OR = 144.975; *p* < 0.001), PSAD (OR = 1501.218; *p* < 0.001), and TZ volume (OR = 0.973; *p* < 0.001) as parameters strongly associated with prostate cancer. On multivariate analysis, high multicollinearity necessitated the evaluation of volumetric and density parameters in separate models. TZ volume emerged as an independent predictor in Model A (aOR = 0.924; *p* < 0.001), while TZ-PSAD was identified as an independent predictor in Model B (aOR = 103.443; *p* < 0.001). PI-RADS ≥ 3 remained an independent predictor in both models (Table 3a).

In the PSA 10–20 ng/mL group, TZ-PSAD demonstrated a strong association on univariate analysis (OR = 322.493; *p* < 0.001) and retained its independent predictive value in multivariate Model B (aOR = 121.021; *p* < 0.001). PI-RADS ≥ 3 was similarly identified as a significant independent predictor in both models (Table 3b).

### 3.5. Stepwise Regression

As a complementary approach, forward stepwise logistic regression confirmed transition zone density as the first and most consistent variable selected in both PSA strata (entry *p* < 0.001 in both groups; Table 3d). In the PSA 4–10 ng/mL group, the highest overall correct classification rate (72.6%; Nagelkerke R^2^ = 0.345) was reached at a five-variable model incorporating transition zone density, age, peripheral zone density, PI-RADS ≥ 3, and DRE Grade 2–3. In the PSA 10–20 ng/mL group, transition zone density alone yielded the highest classification rate (78.7%; Nagelkerke R^2^ = 0.505); the subsequent addition of PSA density reduced the classification rate to 77.5% and was therefore not retained. These findings corroborate the forced-entry Model B reported above (Table 3a,b).

### 3.6. Internal Validation and Sensitivity Analyses

In a pre-specified sensitivity analysis using clinically significant prostate cancer (ISUP Grade Group ≥ 2) as the outcome, TZ-PSAD retained good discrimination in both strata (AUC 0.721 and 0.830, respectively); however, its DeLong-tested superiority over conventional PSA density was no longer statistically significant in the PSA 4–10 ng/mL group (*p* = 0.131), while remaining significant in the PSA 10–20 ng/mL group (*p* = 0.024) (Table 4a). To test whether this signal reflects an incremental density effect or simply transition zone size, TZ-PSAD was additionally compared against TZ volume alone using DeLong’s test: no significant difference was observed in either stratum, for either the any-grade or csPCa outcome (all *p* > 0.07; Table 4b), and a likelihood-ratio test confirmed that adding serum PSA to a model containing TZ volume alone did not improve model fit (*p* = 0.152 and *p* = 0.779). These findings indicate that the diagnostic signal is largely attributable to transition zone size rather than to PSA normalization per se. A biopsy-avoidance analysis at the Youden-optimal TZ-PSAD cut-off showed that deferring biopsy below this threshold would have missed 34.8% of all cancers and 17.7% of clinically significant cancers in the PSA 4–10 ng/mL group, compared with 23.4% and 8.5%, respectively, in the PSA 10–20 ng/mL group; decision-curve analysis of Model B showed net benefit exceeding both treat-all and treat-none strategies across an approximately 20–50% threshold-probability range in both strata (Table 5). Bootstrap internal validation (1000 replicates) of the final multivariable density model (Model B) showed limited optimism in both strata: the apparent AUC of 0.765 in the PSA 4–10 ng/mL group was corrected to 0.757, with a calibration slope of 0.981; the apparent AUC of 0.884 in the PSA 10–20 ng/mL group was corrected to 0.868, with a calibration slope of 1.016 (Table 6).

## 4. Discussion

In this study, TZ-PSAD and conventional PSAD were both significantly associated with prostate cancer in the PSA gray zone, and TZ-PSAD, in combination with PI-RADS ≥ 3, emerged as an independent predictor in both PSA groups. Transition zone volume was likewise significantly associated with prostate cancer and retained independent predictive value in the volumetric model. Conversely, serum PSA levels, PZ-PSAD, and PZ volume failed to show significant differences between BPH and prostate cancer in either group.

The superiority of TZ-PSAD over conventional PSAD was further confirmed by DeLong’s test in both PSA strata; however, this advantage attenuated in the PSA 4–10 ng/mL group when the outcome was restricted to clinically significant cancer (ISUP Grade Group ≥ 2), while remaining significant in the PSA 10–20 ng/mL group, indicating that the comparative advantage of TZ-PSAD is most robust in the higher PSA stratum. Notably, TZ-PSAD showed no significant advantage over TZ volume alone in either stratum, suggesting that its discriminative signal is largely attributable to transition zone size rather than to an independent density effect.

In our study, serum PSA levels failed to demonstrate a statistically significant difference between BPH and prostate cancer in either the PSA 4–10 ng/mL or PSA 10–20 ng/mL group. This finding reaffirms that PSA is an organ-specific rather than a cancer-specific marker and underscores the inadequacy of biopsy decisions based solely on serum PSA in the PSA gray zone. Consistent with this observation, the literature has established that PSA levels within the gray zone offer limited discriminatory capacity between BPH and prostate cancer, contributing to high false-positive rates [5]. These findings highlight the need for more specific complementary parameters to guide biopsy decision-making.

The diagnostic superiority of TZ-PSAD over conventional PSAD has been increasingly recognized in recent years [6,7,8]. Consistent with the literature, TZ-PSAD yielded higher AUC values than PSAD in both PSA groups in the present study, with formal DeLong testing confirming this difference most robustly in the PSA 10–20 ng/mL group.

The PSA range of 4–10 ng/mL is defined as the diagnostic gray zone, within which serum PSA alone does not provide sufficient evidence to guide biopsy decisions and may lead to unnecessary procedures [9,10]. To address this limitation, derived parameters reflecting zone-based PSA-volume relationships, namely TZ-PSAD and PZ-PSAD, have been investigated as complementary tools. Yang L. et al. reported that zone-based PSA density calculations could reduce the rate of unnecessary biopsies by 48% [11]. In parallel, complementary real-time imaging modalities such as micro-ultrasound have also emerged as promising adjuncts for gray-zone risk stratification and prostate cancer staging, and may be used alongside mp-MRI-derived zonal density parameters in future diagnostic algorithms [12]. In our own cohort, however, a biopsy-avoidance analysis at the TZ-PSAD Youden cut-off showed that deferring biopsy below this threshold would have missed 34.8% of all cancers and 17.7% of clinically significant cancers in the PSA 4–10 ng/mL group (versus 23.4% and 8.5%, respectively, in the PSA 10–20 ng/mL group), indicating that the potential for biopsy reduction is considerably more limited in the lower PSA stratum and should be regarded as hypothesis-generating pending prospective, decision-analytic validation.

In our study, TZ-PSAD and PI-RADS ≥ 3 emerged as complementary parameters with independent predictive value in the PSA gray zone, consistent with the results of a validation study by Pausch AM et al. evaluating mp-MRI PI-RADS scores alongside zone-based volumetric parameters [13].

Regarding volumetric differences between groups, total prostate volume and TZ volume were significantly elevated in the BPH group and identified as independent predictors. In contrast, PZ volume did not differ significantly between groups in either PSA stratum, which is consistent with the well-established notion that BPH-related prostatic enlargement predominantly involves the transition zone rather than the peripheral zone. As PZ volume did not increase proportionally with overall prostate size in either group, PZ-PSAD consequently failed to provide meaningful discrimination between prostate cancer and BPH. Multiple studies in the literature have similarly demonstrated the insufficiency of PZ-PSAD for this distinction [6,14].

Nevertheless, conflicting findings exist in the literature. Wang et al. reported PZ-PSAD as a dominant predictor for prostate cancer detection in men with PSA > 4 ng/mL [15]. This discrepancy may be attributable to heterogeneity in study populations, differences in applied PSA cut-off values, and methodological variability in biopsy strategies.

In the present study, the optimal cut-off value for TZ-PSAD was 0.34 ng/mL^2^, consistent with cut-off values reported in the literature [16,17,18]. Sensitivity and specificity, however, differed markedly across PSA groups. In the PSA 4–10 ng/mL group, TZ-PSAD yielded a sensitivity of 44.8% and specificity of 90.8%, whereas these values improved to 73.8% and 94.7%, respectively, in the PSA 10–20 ng/mL group, reflecting a substantial gain in diagnostic reliability. In a comparable study by Shang J. et al., sensitivity was reported as 50% in the PSA 4–10 ng/mL group and 74% in the PSA 10–20 ng/mL group [16]. Despite its high specificity, the sensitivity of TZ-PSAD at the 4–10 ng/mL cut-off remains low, meaning that a substantial proportion of prostate cancers would still be missed if this threshold were applied as a stand-alone triage tool in this PSA range.

In light of these data, TZ-PSAD showed the strongest association with prostate cancer among the density-based parameters evaluated, PSA, PSAD, and PZ-PSAD, although this signal did not significantly exceed that of transition zone volume alone. Given this, along with its relatively low sensitivity in the PSA 4–10 ng/mL group, TZ-PSAD alone is insufficient for reliable differentiation between prostate cancer and BPH and should be evaluated in conjunction with complementary parameters such as the PI-RADS score.

This study has several strengths. Zone-based volumetric measurements obtained through AI-assisted mp-MRI segmentation enabled standardized and reproducible calculation of TZ-PSAD and PZ-PSAD. By stratifying patients into two groups according to PSA level, heterogeneity within the gray zone was controlled and independent predictive parameters were identified separately for each PSA stratum. Furthermore, this study is one of the few to evaluate TZ-PSAD and PI-RADS score jointly within the same multivariate model in the PSA gray zone.

Several limitations of this study warrant consideration. First, the retrospective single-center design precludes complete exclusion of selection bias, and the generalizability of findings to different patient populations remains limited. Second, the relatively small sample size in the PSA 10–20 ng/mL group (*n* = 82) constrains the statistical power of findings in this stratum, necessitating validation in larger cohorts. Third, the sensitivity of 44.8% observed at the optimal TZ-PSAD cut-off in the PSA 4–10 ng/mL group suggests that this parameter alone may be insufficient to guide biopsy decisions in this range. Lastly, the lack of full standardization of AI-assisted segmentation software across different platforms and MRI protocols limits the direct comparability of findings across centers. Before TZ-PSAD can be considered for routine clinical practice, multi-center prospective validation studies with larger patient cohorts are warranted to address these limitations.

## 5. Conclusions

In patients with serum PSA levels of 4–20 ng/mL, TZ-PSAD and conventional PSAD were both significantly associated with prostate cancer, whereas serum PSA and PZ-PSAD were not, and TZ-PSAD, combined with PI-RADS ≥ 3, emerged as an independent predictor in both PSA groups; however, this signal was largely attributable to transition zone size. Zone-specific PSA density, alongside PI-RADS, may have a complementary role in guiding biopsy decisions, pending prospective validation.

## Figures and Tables

**Table 1:** (**a**) Baseline demographic and clinical characteristics, stratified by PSA group and histopathological diagnosis. (**b**) MRI PI-RADS scores, ISUP Grade Groups, and MRI-derived volumetric and PSA density parameters, stratified by PSA group and histopathological diagnosis.

(**a**)						
**Variable**	**PSA Group 4–10 ng/mL**			**PSA Group 10–20 ng/mL**		
	**BPH (*n* = 142)**	**PCa (*n* = 130)**	***p*-Value**	**BPH (*n* = 40)**	**PCa (*n* = 42)**	***p*-Value**
Age, years, median (IQR)	64 (60–69)	66 (61-70)	0.059	66.5 (61.75–70)	65 (62–68.75)	0.759
Comorbidities						
Hypertension	58 (40.8%)	63 (48.5%)	0.254	16 (40%)	22 (52.4%)	0.367
Diabetes mellitus	33 (23.2%)	28 (21.5%)	0.849	12 (30%)	10 (23.8%)	0.702
Coronary artery disease	32 (22.5%)	24 (18.5%)	0.497	11 (27.5%)	3 (7.1%)	0.031
COPD	6 (4.2%)	8 (6.2%)	0.667	3 (7.5%)	1 (2.4%)	0.574
Rectal Examination (DRE) Grade						
Grade 1	48 (33.8%)	71 (54.6%)	**0.001**	10 (25.0%)	23 (54.8%)	**0.020**
Grade 2	77 (54.2%)	43 (33.1%)		20 (50.0%)	14 (33.3%)	
Grade 3	17 (12.0%)	16 (12.3%)		10 (25.0%)	5 (11.9%)	
(**b**)						
**Variable**	**PSA Group 4–10 ng/mL**			**PSA Group 10–20 ng/mL**		
	**BPH (*n* = 142)**	**PCa (*n* = 130)**	***p*-Value**	**BPH (*n* = 40)**	**PCa (*n* = 42)**	***p*-Value**
MRI PI-RADS Score						
PI-RADS 2	32 (22.5%)	11 (8.5%)	<0.001	11 (27.5%)	2 (4.8%)	<0.001
PI-RADS 3	42 (29.6%)	40 (30.8%)		15 (37.5%)	8 (19%)	
PI-RADS 4	63 (44.4%)	55 (42.3%)		11 (27.5%)	13 (31%)	
PI-RADS 5	5 (3.5%)	24 (18.5%)		3 (7.5%)	19 (45.2%)	
ISUP Grade (Prostate Cancer Patients Only)						
Grade 1	—	56 (43.1%)		—	16 (38.1%)	
Grade 2	—	47 (36.2%)		—	13 (31%)	
Grade 3	—	14 (10.8%)		—	6 (14.3%)	
Grade 4	—	9 (6.9%)		—	5 (11.9%)	
Grade 5	—	4 (3.1%)		—	2 (4.8%)	
PSA, ng/mL	6.42 (5.17–8.13)	6.49 (5.45–7.91)	0.526	12.60 (11.57–14.10)	12.45 (10.86–15.20)	0.553
Prostate volume, mL	50.82 (38.25–68.75)	40.00 (29.25–52.75)	<0.001	66.50 (44.84–84.79)	36.25 (27.75–49.33)	<0.001
Peripheral zone volume, mL	14.76 (11.20–18.00)	15.00 (12.00–19.00)	0.441	14.13 (11.72–18.50)	16.62 (11.54–19.05)	0.548
Transition zone volume, mL	39 (25.92–50.1)	22 (13.51–35.47)	<0.001	56 (34.08–70.34)	17.45 (12.42–27)	<0.001
PSA density, ng/mL^2^	0.13 (0.09–0.19)	0.18 (0.12–0.24)	<0.001	0.21 (0.15–0.28)	0.30 (0.19–0.47)	0.006
PZ-PSAD, ng/mL^2^	0.46 (0.33–0.60)	0.43 (0.35–0.59)	0.939	0.79 (0.59–1.08)	0.81 (0.62–1.19)	0.791
TZ-PSAD, ng/mL^2^	0.18 (0.11–0.25)	0.29 (0.17–0.49)	<0.001	0.22 (0.17–0.34)	0.68 (0.41–1.03)	<0.001

IQR, interquartile range; BPH, benign prostatic hyperplasia; PCa, prostate cancer; DRE, digital rectal examination; COPD, chronic obstructive pulmonary disease. *p*-values from Mann–Whitney U test (continuous) and Pearson chi-square test (categorical). This table isolates baseline demographic and clinical variables from imaging/pathology parameters (see Table 1b). PI-RADS, Prostate Imaging Reporting and Data System; ISUP, International Society of Urological Pathology; PZ-PSAD, peripheral zone PSA density; TZ-PSAD, transition zone PSA density. Values are median (IQR) or *n* (%). *p*-values from Mann–Whitney U test and Pearson chi-square test.

**Table 2:** ROC analysis for detection of prostate cancer, with DeLong comparison of correlated ROC curves (TZ-PSAD vs. PSA density).

Variable	PSA Group 4–10 ng/mL				PSA Group 10–20 ng/mL			
	AUC (95% CI)	*p*-Value	Cut-Off	Sens/Spec (%)	AUC (95% CI)	*p*-Value	Cut-Off	Sens/Spec (%)
Prostate Volume	0.661 (0.596–0.726)	<0.001	≤34.5	36.9/88	0.770 (0.668–0.872)	<0.001	≤47.3	73.8/70
TZ Volume	0.713 (0.651–0.776)	<0.001	≤22.37	53.6/83.1	0.820 (0.728–0.912)	<0.001	≤27	76.2/84.6
PSA Density	0.660 (0.595–0.724)	<0.001	≥0.21	40.8/84.5	0.676 (0.560–0.792)	0.003	≥0.41	35.7/97.5
TZ Density	0.711 (0.649–0.774)	<0.001	≥0.34	44.8/90.8	0.841 (0.754–0.928)	<0.001	≥0.48	73.8/94.7

AUC, area under the curve; CI, confidence interval; Sens, sensitivity; Spec, specificity; TZ, transition zone. Optimal cut-off determined by Youden index (sensitivity + specificity − 1). Confidence intervals calculated using the Hanley–McNeil method. For prostate volume and TZ volume (inverse predictors of PCa), AUC reflects discriminative performance after directional inversion; cut-off denotes the threshold below which PCa is predicted (≤). *p*-values reflect comparison of AUC against the null hypothesis of AUC = 0.5.

**Table 3:** (**a**) Final multivariable models for prostate cancer detection—PSA 4–10 ng/mL group (*n* = 2), Firth penalized logistic regression. (**b**) Final multivariable models for prostate cancer detection—PSA 10–20 ng/mL group (*n* = 82), Firth penalized logistic regression. (**c**) Univariate screening—all candidate predictors (Firth logistic regression, rescaled units). (**d**) Forward stepwise logistic regression (likelihood-ratio entry criterion, *p* < 0.05).

(**a**)					
**Variable**		**Multivariate Model A** **(Volumetric)**		**Multivariate Model B** **(Density)**	
		**aOR (95% CI)**	***p*-Value**	**aOR (95% CI)**	***p*-Value**
Age, per year		1.058 (1.016–1.101)	0.006	—	—
TZ volume, per 10 mL decrease		0.776 (0.683–0.882)	<0.001	—	—
TZ-PSAD, per 0.1 ng/mL^2^ increase		—	—	1.567 (1.313–1.871)	<0.001
DRE Grade 2–3 (vs. Grade 1)		0.545 (0.319–0.931)	0.026	0.512 (0.298–0.882)	0.016
PI-RADS ≥ 3 (vs. PI-RADS 2)		3.023 (1.404–6.511)	0.005	3.011 (1.350–6.719)	0.007
(**b**)					
**Variable**		**Multivariate Model A** **(Volumetric)**		**Multivariate Model B** **(Density)**	
		**aOR (95% CI)**	***p*-Value**	**aOR (95% CI)**	***p*-Value**
TZ volume, per 10 mL decrease		0.721 (0.585–0.888)	0.002	—	—
TZ-PSAD, per 0.1 ng/mL^2^ increase		—	—	1.614 (1.247–2.090)	<0.001
DRE Grade 2–3 (vs. Grade 1)		0.469 (0.152–1.445)	0.187	0.629 (0.183–2.165)	0.462
PI-RADS ≥ 3 (vs. PI-RADS 2)		6.111 (1.175–31.792)	0.031	5.355 (0.837–34.262)	0.076
(**c**)					
**Variable (Unit)**		**PSA 4–10 ng/mL**		**PSA 10–20 ng/mL**	
		**OR (95% CI)**	***p***	**OR (95% CI)**	***p***
Age (per year)		1.043 (1.004–1.083)	0.028	0.984 (0.919–1.054)	0.651
Serum PSA (per ng/mL)		1.044 (0.896–1.216)	0.584	1.024 (0.841–1.248)	0.810
Prostate volume (per 10 mL)		0.827 (0.744–0.920)	<0.001	0.739 (0.613–0.891)	0.002
PZ volume (per 10 mL)		1.103 (0.714–1.705)	0.658	0.990 (0.490–2.000)	0.977
TZ volume (per 10 mL)		0.768 (0.679–0.868)	<0.001	0.661 (0.533–0.820)	<0.001
PSA density (per 0.1 ng/mL^2^)		2.064 (1.488–2.863)	<0.001	1.737 (1.202–2.511)	0.003
PZ-PSAD (per 0.1 ng/mL^2^)		0.984 (0.898–1.080)	0.740	1.023 (0.925–1.132)	0.660
TZ-PSAD (per 0.1 ng/mL^2^)		1.624 (1.360–1.939)	<0.001	1.714 (1.322–2.221)	<0.001
Hypertension (yes vs. no)		1.277 (0.786–2.076)	0.324	1.664 (0.685–4.045)	0.261
Diabetes mellitus (yes vs. no)		0.985 (0.553–1.752)	0.959	0.773 (0.285–2.095)	0.612
Coronary artery disease (yes vs. no)		0.772 (0.424–1.407)	0.399	0.241 (0.063–0.924)	0.038
COPD (yes vs. no)		1.508 (0.509–4.466)	0.458	0.367 (0.041–3.261)	0.368
DRE Grade 2–3 (vs Grade 1)		0.408 (0.249–0.670)	<0.001	0.267 (0.102–0.697)	0.007
PI-RADS ≥ 3 (vs PI-RADS 2)		2.955 (1.425–6.127)	0.004	6.775 (1.510–30.388)	0.012
(**d**)					
**Step**	**Variable Entered**	**Entry *p***	**Overall CCR**	**Nagelkerke R^2^**	**Note**
		**PSA 4–10 group**			
1	TZ-PSAD	<0.001	0.673	0.212	PSA 4–10 group
2	+ Age	0.001	0.680	0.255	
3	+ PZ-PSAD	0.002	0.699	0.293	
4	+ PI-RADS ≥ 3	0.003	0.707	0.328	
5	+ DRE Grade 2–3	0.033	0.726	0.345	Final model (highest CCR)
		**PSA 10–20 group**			
1	TZ-PSAD	<0.001	0.787	0.505	Final model (highest CCR)
2	+ PSA density	0.019	0.775	0.560	CCR decreased; not retained

aOR, adjusted odds ratio; CI, confidence interval; TZ, transition zone; TZ-PSAD, transition zone PSA density; DRE, digital rectal examination; PI-RADS, Prostate Imaging Reporting and Data System. Both models were fitted using Firth penalized (bias-reduced) logistic regression to address near-separation in the small-sample density model. Continuous predictors were rescaled to clinically interpretable increments (per 0.1 ng/mL^2^ for density parameters, per 10 mL for volume, per year for age); this resolves the uninterpretable odds ratios reported in the original submission (e.g., PSA density aOR 1501, TZ-PSAD aOR 103), which were an artifact of per-unit scaling of a fractional variable. Model A was simplified to a single volumetric predictor (TZ volume) because total prostate volume and TZ volume are highly collinear (Spearman ρ = 0.92) and could not be estimated stably together; PSA density remains excluded from Model B for the same reason relative to TZ-PSAD (ρ = 0.80). Internal validation of Model B (bootstrap, B = 1000) is reported in the main text and Table 6: optimism-corrected AUC 0.757, calibration slope 0.981. Abbreviations as in Table 3a. In this smaller stratum (*n* = 80, 42 events), DRE Grade and PI-RADS lose statistical significance once entered jointly with density/volumetric predictors in the Firth-adjusted model, despite significance on univariate screening (Table 3c)—reflecting the limited power of this subgroup rather than a data inconsistency. TZ-PSAD retains independent predictive value in both PSA strata. Internal validation of Model B: optimism-corrected AUC 0.868, calibration slope 1.016 (Table 6). OR, odds ratio; CI, confidence interval; PZ, peripheral zone; TZ, transition zone; DRE, digital rectal examination; PI-RADS, Prostate Imaging Reporting and Data System; COPD, chronic obstructive pulmonary disease. All models fitted by Firth penalized logistic regression, one predictor at a time. This table replaces the univariate columns previously embedded in Table 3, isolating exploratory screening from the pre-specified final models. CCR, overall correct classification rate; TZ-PSAD, transition zone PSA density; PZ-PSAD, peripheral zone PSA density; DRE, digital rectal examination; PI-RADS, Prostate Imaging Reporting and Data System. Per the model-selection rule stated in Methods (Section 2.4), the step with the highest CCR was selected as final: a 5-variable model in the PSA 4–10 ng/mL group, and TZ-PSAD alone in the PSA 10–20 ng/mL group (adding PSA density at step 2 reduced CCR from 0.787 to 0.775 and was therefore not retained). TZ-PSAD was the first and most consistent variable to enter in both strata, corroborating the forced-entry Model B in Table 3.

**Table 4:** (**a**) Sensitivity analysis using clinically significant prostate cancer (ISUP Grade Group ≥ 2) as the outcome. (**b**) TZ-PSAD versus transition zone volume alone (test for a gland-size effect).

(**a**)				
**PSA Group**	**csPCa *n* (%)**	**AUC PSA Density** **(95% CI)**	**AUC TZ-PSAD** **(95% CI)**	**DeLong *p*** **(PSAD vs. TZ-PSAD)**
4–10 ng/mL (*n* = 267)	71 (26.6%)	0.686 (0.617–0.756) *	0.721 (0.652–0.789) *	0.131
10–20 ng/mL (*n* = 80)	26 (32.5%)	0.723 (0.601–0.845) *	0.830 (0.727–0.933) *	0.024
(**b**)				
**PSA Group**	**Outcome Definition**	**AUC TZ-PSAD** **(95% CI)**	**AUC TZ Volume** **(95% CI)**	**DeLong *p***
4–10 ng/mL	Any-grade PCa (primary)	0.711 (0.649–0.774)	0.713 (0.651–0.776)	0.856
4–10 ng/mL	csPCa (ISUP ≥ 2)	0.721 (0.647–0.794)	0.697 (0.622–0.772)	0.071
10–20 ng/mL	Any-grade PCa (primary)	0.841 (0.754–0.928)	0.841 (0.755–0.928)	0.980
10–20 ng/mL	csPCa (ISUP ≥ 2)	0.830 (0.725–0.935)	0.858 (0.760–0.955)	0.073

csPCa, clinically significant prostate cancer, defined as ISUP Grade Group ≥ 2 (ISUP Grade Group 1 reclassified with BPH as the non-significant reference category), per Reviewer 2 (R2-1). * Hanley–McNeil 95% CI. TZ-PSAD retains good discrimination for csPCa in both strata (AUC 0.72 and 0.83), and both TZ-PSAD and PSA density remain significantly higher in csPCa than in the non-significant group (Mann–Whitney *p* < 0.001 in both strata). However, the formal superiority of TZ-PSAD over conventional PSA density (DeLong test) is no longer statistically significant in the PSA 4–10 ng/mL group once the outcome is restricted to csPCa (*p* = 0.131), while it remains significant in the PSA 10–20 ng/mL group (*p* = 0.024). AUC, area under the curve; TZ, transition zone; csPCa, clinically significant prostate cancer (ISUP Grade Group ≥ 2). DeLong test for two correlated ROC curves (TZ-PSAD vs. TZ volume, same patients). Under the primary outcome (any-grade PCa), TZ-PSAD and TZ volume alone are statistically indistinguishable in both PSA strata (*p* = 0.856 and *p* = 0.980); under the csPCa outcome, the two remain non-significantly different in both strata (*p* = 0.071 and *p* = 0.073). A likelihood-ratio test confirmed that adding serum PSA to a model containing TZ volume alone did not significantly improve model fit in either stratum (χ^2^ = 2.056, *p* = 0.152 for PSA 4–10 ng/mL; χ^2^ = 0.079, *p* = 0.779 for PSA 10–20 ng/mL). Taken together, these results indicate that the diagnostic signal previously attributed to TZ-PSAD is statistically attributable to transition zone gland size rather than to PSA normalization per se; serum PSA does not add discriminative information once TZ volume is known.

**Table 5:** Biopsy-avoidance analysis at the TZ-PSAD Youden cut-off.

PSA Group/Cut-Off	Biopsy Indicated (≥Cut-Off), *n*	Biopsy Avoided (<Cut-Off), *n*	Any PCa Missed Among Avoided, *n* (%)	csPCa (ISUP ≥ 2) Missed Among Avoided, *n* (%)
4–10 ng/mL, TZ-PSAD ≥0.34	69 (TP 56/FP 13)	198 (TN 129/FN 69)	69/198 (34.8%)	35/198 (17.7%)
10–20 ng/mL, TZ-PSAD ≥0.48	33 (TP 31/FP 2)	47 (TN 36/FN 11)	11/47 (23.4%)	4/47 (8.5%)

TP, true positive; FP, false positive; TN, true negative; FN, false negative; csPCa, clinically significant prostate cancer (ISUP Grade Group ≥ 2). At the Youden-optimal TZ-PSAD cut-off, biopsy avoidance in the PSA 4–10 ng/mL group would miss a substantial proportion of cancers, including 17.7% clinically significant cases—too high a miss rate to responsibly support biopsy deferral based on TZ-PSAD alone in this stratum. Performance is more favorable in the PSA 10–20 ng/mL group (8.5% csPCa missed). Decision-curve analysis of Model B (density model) showed net benefit exceeding both treat-all and treat-none strategies across the clinically plausible threshold-probability range (~20–50%) in both strata.

**Table 6:** Internal validation of Model B (density model) by bootstrap resampling (B = 1000).

PSA Group	Apparent AUC	Optimism-Corrected AUC	Calibration Slope (Shrinkage Factor)
4–10 ng/mL	0.765	0.757	0.981
10–20 ng/mL	0.884	0.868	1.016

AUC, area under the curve. Optimism was estimated as the mean difference between bootstrap-model performance on the bootstrap sample and on the original sample (Harrell’s bootstrap optimism method), based on Model B (TZ-PSAD + DRE Grade + PI-RADS ≥ 3), fitted by Firth penalized logistic regression at each replicate. The small optimism (0.008–0.016 AUC units) and near-unity calibration slopes indicate limited overfitting and support the internal robustness of Model B.

## Data Availability

The data that support the findings of this study are not publicly available due to patient privacy and ethical restrictions, but are available from the corresponding author upon reasonable request.
